# P-1129. Microbiologically-defined Tissue Expander Infections: Evaluating risk factors for recurrence and optimal management strategies

**DOI:** 10.1093/ofid/ofaf695.1323

**Published:** 2026-01-11

**Authors:** Rita Igwilo-Alaneme, Nischal Ranganath, Aparna Vijayasekaran, Allison LeMahieu, nishant Kumar, Vasupriya Ravi, Muhammad Sabry Mazroua, Ryan W W Stevens, Aditya Shah

**Affiliations:** Mayo Clinic, Rochester, Minnesota; Mayo Clinic, Rochester, Minnesota; Mayo Clinic Rochester, Rochester, Minnesota; Mayo Clinic Rochester, Rochester, Minnesota; Mayo Clinic Rochester, Rochester, Minnesota; Mayo Clinic Rochester, Rochester, Minnesota; Mayo Clinic Rochester, Rochester, Minnesota; Mayo Clinic, Rochester, Minnesota; Mayo Clinic, Rochester, Minnesota

## Abstract

**Background:**

In 2019, the US performed 290,000 breast reconstructions with an estimated infection rate of 3-35%. Tissue expander infections detract from desired aesthetic effects, delay adjuvant treatments, increase morbidity and mortality, and have high recurrence rates (20-40%). Little is known about risk factors for recurrent tissue expander infections, particularly the impacts of different treatment modalities.

**Figure:**
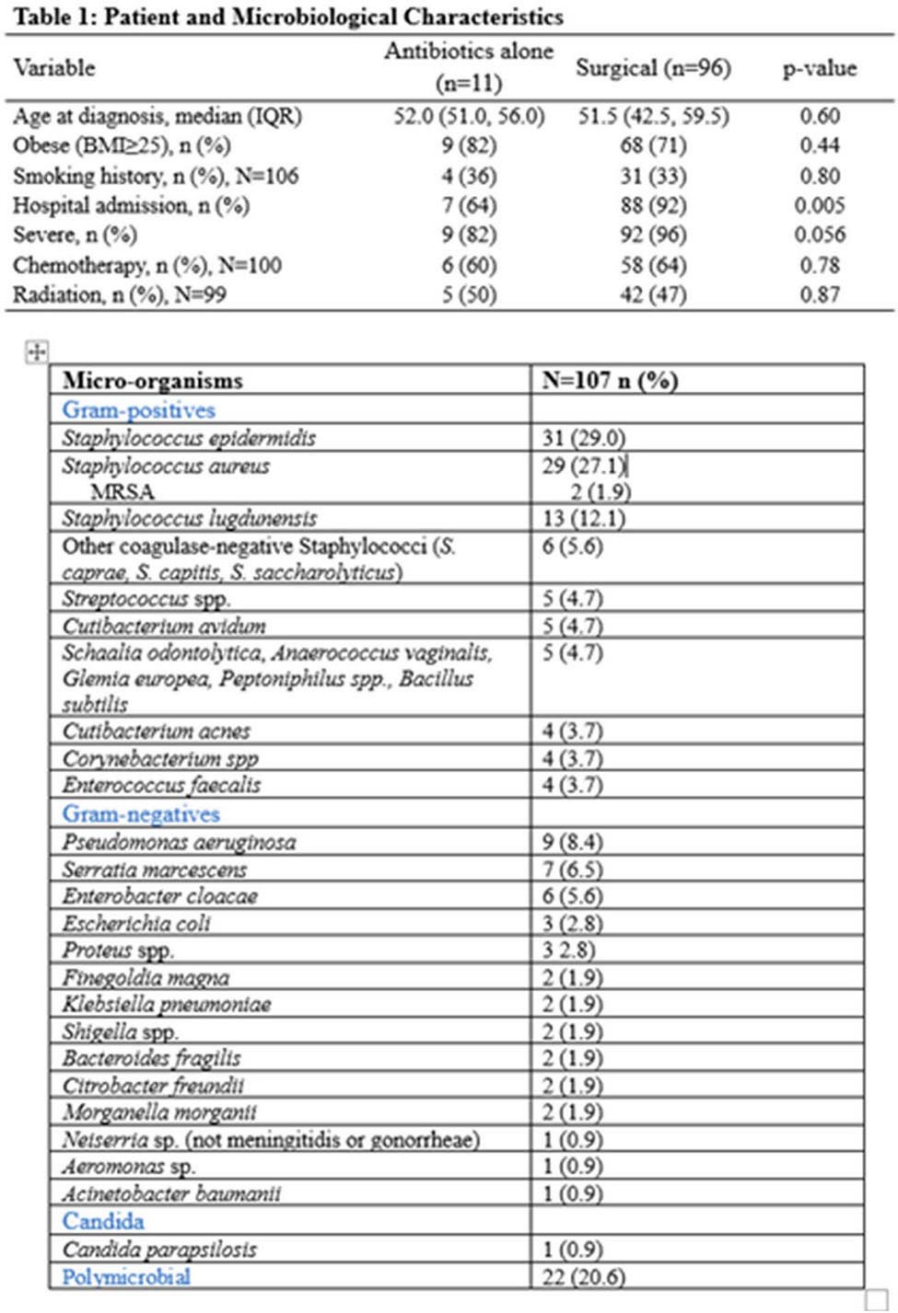

**Figure:**
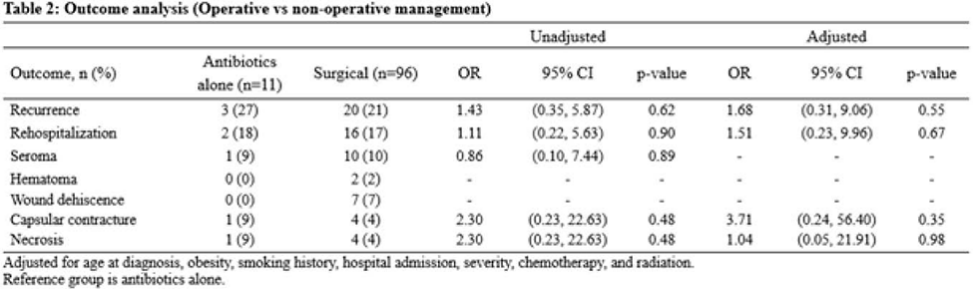

**Methods:**

We conducted a single-center retrospective cohort study of adult women post-mastectomy who developed microbiologically-proven tissue expander infection between the period of 2017 – 2024. Outcomes including 12-month infection recurrence and risk factors for recurrence were compared between surgical versus nonoperative groups, as well as between three surgical strategies (i.e., one-stage, two-stage, explantation). Unadjusted and adjusted logistic regression models were used to assess the associations between the treatment modalities and the outcomes. Univariate models assessed the relationship between the covariates of interest and infection recurrence.

**Figure:**
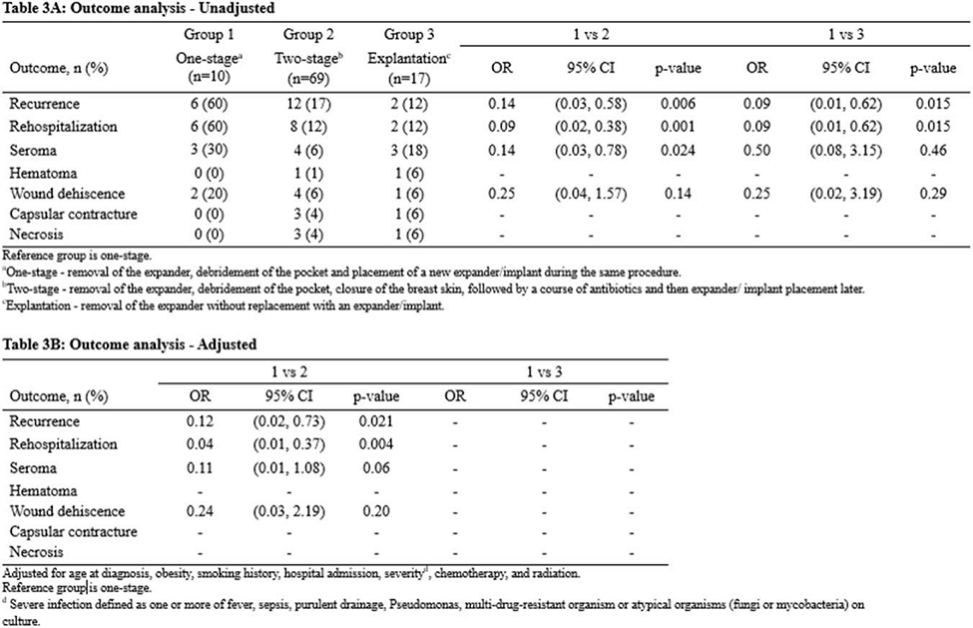

**Figure:**
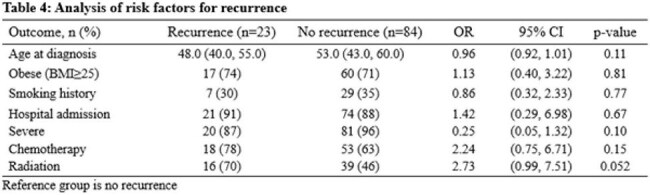

**Results:**

107 patients were included, with the majority treated with combination of antibiotics and surgical therapy (Table 1). There was no significant difference in outcomes when the operative versus antibiotics alone cohorts were compared (Table 2). The odds of infection recurrence and rehospitalization in patients who underwent two-staged exchange or explantation were significantly lower when compared with the one-staged group (Table 3). Univariate modeling found severity and radiation therapy to be numerically more common in the group experiencing recurrence, with a trend towards statistical significance (Table 4).

**Conclusion:**

Our study demonstrates reduced risk of recurrent tissue expander infection with two-staged exchange or explantation, compared to one-staged exchange. Though limited by small sample size, the strengths of our study include our presentation of the real-world variability of these syndromes and their management, adjustment for infection severity and other potential confounders; and the focus on microbiologically-defined infections.

**Disclosures:**

All Authors: No reported disclosures

